# Examining the Association Between Referral Quality, Wait Time and Patient Outcomes for Patients Referred to an IBD Specialty Program

**DOI:** 10.1093/jcag/gwz002

**Published:** 2019-02-20

**Authors:** Holly Mathias, Courtney Heisler, Julia Morrison,, Barbara Currie, Kelly Phalen-Kelly, Jennifer Jones

**Affiliations:** 1 Nova Scotia Health Authority, Centre for Clinical Research, Halifax, Nova Scotia, Canada; 2 Nova Scotia Collaborative IBD Program, Division of Digestive Care and Endoscopy, QEII Health Sciences Centre, Halifax, Nova Scotia, Canada

**Keywords:** Inflammatory bowel disease, Referral and consultation, Quality Improvement, Wait time

## Abstract

**Background:**

Most speciality inflammatory bowel disease (IBD) care can only be accessed through a referral. Timely access to specialty care has been associated with improved disease-related outcomes. To receive appropriate care, the referral needs to include high-quality information. To date, no research has explored the association between referral quality and IBD patient outcomes. The study objectives were to determine if the quality of referrals to a collaborative IBD program influenced triage accuracy, wait times and patient outcomes.

**Methods:**

Two hundred referrals to a collaborative IBD program in Canada for patients with confirmed or suspected IBD were reviewed. Referral quality was evaluated using an evidence- and consensus-based metric. The association between referral quality and patient outcomes (wait time, hospitalizations, disease flares and additional referrals) for semi-urgent referrals was assessed through multivariate analysis.

**Results:**

The majority of referrals for IBD speciality care were categorized as being low quality. Referral quality was not significantly associated with any of the patient outcomes; however, longer wait times significantly increased the occurrence of disease flares, hospitalizations and additional referrals while waiting for an IBD specialist appointment.

**Conclusion:**

Prolonged wait times for IBD patients are significantly associated with poor patient outcomes and increased costs for the health care system. Although there is literature that suggests that referral quality may be associated with wait time, it is still unclear how it relates to wait time and patient outcomes. Moving forward, the current referral process needs to be critically addressed in order to improve wait times and patient outcomes.

Inflammatory bowel disease (IBD) is an immune-mediated disease, which includes Crohn’s disease and ulcerative colitis. The etiology of IBD is unknown; however, due to its early onset, IBD can result in high morbidity and, in some cases, mortality (1). The disease also carries a large socioeconomic burden, resulting in missed work and opportunities, and lost productivity for patients, as well as increased economic burdens on health care systems globally (2–4). Increased attention has been given to IBD as the global incidence rate increases, with Canada having the highest rate of IBD in the world (5–8). Because of the physical, mental, social and economic ramifications of IBD, early recognition of the disease and timely referral to a specialist is crucial. Past research has indicated that IBD patient outcomes improve with access to IBD speciality care (9).

In Canada, speciality care can only be accessed through a referral. In most cases, a general practitioner or family doctor sends the referral; however, other physicians, such as surgeons or those in the emergency department, may also refer. It is important that the referral contains all pertinent information for appropriate patient triage and delivery of timely health care services (10). The Canadian Association of Gastroenterology suggests a wait time of less than 2 weeks for patients with clinical symptoms of active IBD (11,12).

Historically, physician surveys have indicated a lack of informative referral communication. Referrals are often returned to the referring physicians for additional information, which is costly to the patient, as well as the physicians involved (10,13,14). A 2012 survey found that approximately two-thirds of specialist physicians reported a lack of basic or supporting information (e.g., relevant lab test results or even reason for referral) (10). Furthermore, there is often variation among specialists, even in the same specialty, in terms of acceptable referrals, which creates inefficiencies, as the referring physicians must figure out what information each individual specialist requires. Additional studies in the field of gastroenterology (GI) have also noted a lack of information cited on referrals; however, no published work has addressed how referral quality may influence patient outcomes (14,15)

## MATERIALS AND METHODS

### Objectives and Research Questions

The main objective of the study was to determine if referrals to the Nova Scotia Collaborative Inflammatory Bowel Disease (NSCIBD) program for patients with suspected or confirmed IBD contained sufficient clinical information to allow accurate triage for timely access to subsequent outpatient care (Question 1: Is there an association between referral quality and patient wait time from initial referral and initial specialist consultation?). The secondary objective of this study was to determine how the quality of initial referrals to the NSCIBD program informed additional patient outcomes during the time between initial referral and first appointment with an IBD specialist (Question 2: Is there an association between referral quality and patient outcomes, such as disease flares, hospitalizations and additional referrals?).

### Referral Process

The NSCIBD program, located on the east coast of Canada in Halifax, Nova Scotia, strives for excellence in patient-centered IBD care (16). When this study was conducted, the referral process to the NSCIBD program was a centralized referral system for all general gastroenterology referrals. Each triage category is defined by specific clinical criteria. Urgent referrals were immediately assigned to a specific gastroenterologist with the expectation that the patient will be seen within 2 weeks. Semi-urgent and nonurgent referrals are placed on a pooled waitlist to be assigned and distributed in order.

### Study Design

This study was a retrospective cohort study included 200 patient referrals from referring health providers (i.e., general practitioners, gastroenterologists, surgeons, nurse practitioners) for individuals who had specialist gastroenterology appointments with the NSCIBD program between August 2016 and December 2017. Referrals were obtained from the electronic medical record and referral quality analyzed between May and December 2017. Referrals were included in the study if they were for a patient’s first visit to the NSCIBD program. Referrals were excluded if they were not for an IBD diagnosis or an IBD-related concern, if the referral was for an endoscopic test, or if the referral was for a follow-up visit.

### Data Collection

Referrals were evaluated using a quality metric and classified as either low, moderate or high quality. The metric was developed in consultation with the specialist clinical team with the NSCIBD program, including a luminal gastroenterologist and two GI nurse practitioners, which was evidence-based and expert/consensus driven (see Chart, Supplementary Data Content 1, for metric used to collect data). The metric was also compared to existing gastroenterology referral metrics and based on the evidence that selected clinical factors may be predictors of IBD disease prognosis (14,15,17,18) and therefore should influence triage urgency classifications. The metric was piloted by the research team to ensure all necessary data could be collected using the form. Referrals were classified as being high, moderate or low quality depending on the information on the metric that was included.

After referral information was captured, patient charts were followed until their first specialist appointment to the NSCIBD program for relevant disease-related outcomes. Relevant patient outcomes during the follow-up period, including disease flares (record of IBD-related symptoms during wait time as reported by patient), hospitalizations, additional referrals and wait time (defined as the time between the date of the initial referral to the NSCIBD program and the time of first IBD speciality appointment) were recorded. The association between referral quality and wait time, number of IBD-related hospitalizations and number of additional referrals to the IBD specialist between the time of initial referral and time of initial specialist consultation was evaluated. Any patient whose medical record was missing information was excluded from the analysis.

### Sample Size and Statistical Analysis

Based on a clinically relevant difference in wait time of 2 weeks and a known mean wait time of 6 months for semi-urgent referrals to the NSCIBD program, it was determined that a minimum of 134 referrals were needed to generate a power of 0.80 given an alpha level of 0.05. A total of 200 referrals were evaluated to accommodate any incomplete medical records or other issues which would result in incomplete data. The moderate- and high-quality categories were collapsed because there were so few high-quality referrals (i.e., <5%). Patient, disease and referral characteristics were analyzed using descriptive statistics. Analysis of variables was descriptive with categorical data summarized as proportions, and ordinal data as means with range and standard deviation. To calculate wait time, the number of weeks between the date of initial referral and the date of initial consultation with a specialist was calculated. T-tests were used to detect statistically significant differences between the groups (low-quality and moderate–high-quality referrals).

Multivariate linear regression was used to evaluate the association between referral quality category (low and moderate–high) and wait time (measured in number of weeks). Logistic regression was used to measure the association between referral quality and disease flares, IBD-related hospitalizations and additional referrals. Only the semi-urgent referrals (*n* = 122), as triaged by the IBD specialty program, were used in order to eliminate the confounding effect of triage classification. We adjusted for the following covariates: referring provider, health region, presence of diagnosis, duration of disease, disease activity, history of resective surgery and indication of current IBD-related medication. We also included wait time as a covariate in the logistic regression analysis. Statistical analyses were conducted using SPSS software (19).

### Ethical Considerations

This study received full approval from the Nova Scotia Health Authority Research Ethics Board.

## RESULTS

### Descriptive Data

#### Administrative referral characteristics

Of the 200 referrals which were analyzed, 159 (79.5%) referrals were considered to be low quality and 41 (20.5%) considered to be moderate–high quality. Only 15 (7.5%) of the referrals were submitted on the standard general GI referral form. Further, 14 (7%) of the referrals were illegible. Over one-half (*n* = 103, 51.5%) of the referrals were from general practitioners and about a quarter (*n* = 49, 24.5%) of the referrals were from gastroenterologists. The remaining referrals were from the emergency department, surgeons, internists, nurse practitioners and other referring practitioners. Table 1 presents the administrative referral characteristics based on how many referrals in total included selected criteria and how many moderate–high- and low-quality referrals included each characteristic.

**Table 1.** Administrative referral characteristics

Characteristics	Total number of referrals which included characteristics (*n*) (%)	Total number of moderate– high-quality referrals which included characteristics (*n*) (%)	Total number of low-quality referrals which included characteristics (*n*) (%)
Standard Form	15 (7.5%)	2 (4.9%)	13 (8.2%)
Legibility	186 (93%)	40 (97.6%)	146 (91.8%)
Referring Provider:			
i) General Practitioner	103 (51.5%)	14 (34.1%)	89 (56%)
ii) Surgeons	12 (6%)	1 (2.4%)	11 (6.9%)
iii) Internists	9 (4.5%)	—	9 (5.7%)
iv) GI	49 (24.5%)	23 (56.1%)	26 (16.4%)
v) Nurse Practitioner	4 (2%)	1 (2.4%)	3 (1.9%)
vi) Emergency Department	22 (11%)	2 (4.9%)	20 (12.6%)
vii) Other	1 (0.5%)	—	1 (0.6%)
Total	200 (100%)	41 (100%)	159 (100%)
Geographic Location of Referring Provider:			
i) In same zone as program	166 (83%)	30 (73.2%)	136 (85.5%)
ii) Outside of program zone, but in same province	20 (10%)	6 (4.6%)	14 (8.8%)
iii) Out of province	14 (7%)	5 (12.2%)	9 (5.7%)
Requested Triage Urgency:			
i) Urgent	26 (13%)	5 (12.2%)	21 (13.2%)
ii) Semi-urgent	16 (8%)	5 (12.2%)	11 (6.9%)
iii) Surveillance	4 (2%)	—	4 (2.5%)
iv) Unknown	154 (77%)	31(75.6%)	123 (77.4%)
Actual Triaged Urgency:			
i) Urgent	35 (17.5%)	9 (22%)	26 (16.4%)
ii) Semi-urgent	122 (61%)	27 (65.9%)	95 (59.7%)
iii) Surveillance	13 (6.5%)	—	13 (8.2%)
iv) Unknown	30 (15%)	5 (12.2%)	25 (15.7%)

#### Clinical characteristics

The majority of referrals to the NSCIBD program included a diagnosis (*n* = 160, 80%); however, supporting information was missing for many of the referrals. Over one-half of the referrals (*n* = 110, 55%) included duration of the disease. Very few referrals provided information relating to the results of investigations. Fifty-two per cent (*n* = 104, 52%) of the referrals provided information on past endoscopies and only 81 (40.5%) referrals provided information on the results of past cross-sectional imaging that may indicate disease extent and phenotype. The majority of the referrals did not provide information on the location, extent or behaviour of the disease. Forty-four per cent (88) of the referrals reported disease location, 58 (29%) reported extent of disease and 57 (28.5%) reported IBD disease behaviour. Only 40 (20%) of the referrals included information about past medical therapies. Table 2 presents the distribution of clinical characteristics on incoming referrals.

**Table 2.** Clinical referral characteristics

Characteristics	Total number of referrals which included characteristics (*n*) (%)	Total number of moderate– high-quality referrals which included characteristics (*n*) (%)	Total number of low- quality referrals which included characteristics (*n*) (%)
Diagnosis	160 (80%)	40 (97.6%)	120 (75.5%)
Duration of disease	111 (55.5%)	40 (97.6%)	71 (44.7%)
Past investigations:			
i) endoscopies	104 (52%)	33 (79.5%)	71 (44.6%)
ii) cross-sectional imaging	81 (40.5%)	39 (96.1%)	42 (26.4%)
iii) IBD-related surgery	113 (56.5%)	36 (87.8%)	72 (45.3%)
Disease severity (current symptoms)	143 (71.5%)	38 (92.7%)	105 (66%)
Disease phenotype	59 (29.5%)	30 (73.1%)	29 (18.2%)
Past medical therapy^*^	40 (20%)	9 (22%) (missing n = 1)	31 (19.5%) (missing n = 54)
Current medical therapy	54 (27%)	15 (36.6%)	39 (24.5%)

*IBD, Inflammatory bowel disease.*

* *n* = 55 missing values concerning past medical therapy.

### Multivariate Analysis and Significance Calculations

#### Referral quality and wait time

Patients referred to the NSCIBD program had a mean wait time of 26.4 weeks (SD = 31.3, range = 0 to 160). Patients who were referred with a low-quality referral had an average wait time of 29.0 weeks (SD = 33.8, range = 0 to 160). Patients who were referred on a moderate–high-quality referral had an average wait time of 16.7 weeks (SD = 14.9, range = 0 to 64). Wait time between low-quality and moderate–high-quality referrals was found to be significantly different (*t*(150.13) = 3.47, *P* < 0.01, confidence interval = 5.29 to 19.32). Univariate analysis found referral quality to be significantly associated with wait time (*t*(198) = 2.27, *P* = 0.01). However, multivariate regression analysis revealed that referral quality was not significantly associated with wait time. Only referring provider (*P* = 0.001) was a significant predictor of wait time (*F*(7) = 3.4, *P* < 0.002, *R*^2^ = 0.17).

### Referral Quality and Patient Outcomes

#### Disease flares

While waiting for specialist IBD care, 7.5% (*n* = 15) of all patients experienced a disease flare while waiting for their IBD specialist appointment (*M* = 0.08, SD = 0.26, range = 0 to 1). After regression analysis, referral quality was not significantly associated with disease flares, although it was a variable included in the final model. Wait time (*P* = 0.019) was the only significant predictor of disease flare (x^2^(3) = 8.03, *P* = 0.046). Semi-urgent patients with a longer wait time were more likely to experience a disease flare.

### IBD-Related Hospitalizations

A total of 23 patients (11.3% of total referrals, *M* = 0.15, SD = 0.44, range = 0 to 3) were hospitalized for IBD-related issues. Five of these patients were hospitalized multiple times. Logistic regression showed that referral quality was not significantly associated with increased IBD-related hospitalizations during wait time, although it was included in the final model. Wait time (*P* = 0.002) was the only significant predictor of IBD-related hospitalizations (x^2^(9) = 14.93, *P* = 0.037). Semi-urgent patients with a longer wait time were 1.05 times more likely to be hospitalized for an IBD-related issue while waiting for their IBD specialist appointment.

#### Additional referrals

There were 41 additional referrals (i.e., referrals which were not requested or solicited by the NSCIBD program) made during patients’ wait time and all were made on behalf of the patients who were initially referred using a low-quality referral (mean = 0.07, SD = 0.26, range = 0 to 1). No one with a moderate–high-quality referral was referred an additional time. After multivariate analysis, referral quality was not found to be associated with additional referrals; however, health region (*P* = 0.048) and wait time (*P* = 0.014) were both significant predictors of additional referrals (x^2^(9) = 25.69, *P* = 0.002). Semi-urgent patients with a longer wait time were 1.04 times more likely to be referred to the IBD specialist program an additional time. A summary table of the output from all regression models can be found in Table 3–6.

**Table 3.** Linear regression analysis for referral quality versus wait time (weeks)

Variable	Coefficient	*P*	CI (95%)
Referring Provider	−3.66	0.001	−5.88–−1.44
Health Region	0.73	0.65	−2.48–3.94
Diagnosis	7.64	0.06	−0.43–15.70
Duration of Disease	−4.81	0.27	−13.40–3.78
Disease Activity	6.87	0.15	−2.50–16.24
Resective Surgery	7.76	0.13	−2.39–17.91
Referral Quality	1.87	0.72	−8.23–11.96

*CI, Confidence interval.*

**Table 4.** Logistic regression analysis for referral quality versus number of disease flares

Variable	Coefficient	*P*	CI (95%)
Referral Quality	1.21	0.25	0.44–25.54
Wait Time (Weeks)	0.04	0.02	1.01–1.07
Resective Surgery	−18.35	`1.00	0.00

*CI, Confidence interval.*

**Table 5.** Logistic regression analysis for referral quality versus number of IBD-related hospitalizations

Variable	Coefficient	*P*	CI (95%)
Health Region	−0.15	0.68	0.43–1.74
Diagnosis	0.21	0.78	0.27–5.58
Duration of Disease	0.26	0.77	0.23–7.20
Disease Activity	−2.18	`0.11	0.01–1.65
Resective Surgery	0.51	0.55	0.31–8.86
Referral Quality	0.45	0.61	0.28–8.86
Wait Time (Weeks)	0.05	0.002	1.02–1.08

*IBD, Inflammatory bowel disease.*

**Table 6.** Logistic regression analysis for referral quality versus number of additional referrals to IBD specialist

Variable	Coefficient	*P*	CI (95%)
Referring Provider	−0.67	0.23	0.17–1.54
Health Region	0.87	0.48	1.01–5.62
Diagnosis	−0.71	0.45	0.08–3.11
Duration of Disease	0.43	0.64	0.25–9.43
Disease Activity	0.65	0.48	0.32–11.55
Resective Surgery	−2.16	0.09	0.01–1.41
IBD-related medication	0.50	0.67	0.16–16.74
Referral Quality	−19.28	1.00	0.00
Wait Time (Weeks)	0.04	0.01	1.01–1.08

*IBD, Inflammatory bowel disease.*

## DISCUSSION

The findings of this study suggest that the majority of referrals received by this IBD speciality program were low quality. Although the multivariate analyses of semi-urgent referrals indicated that referral quality was not a significant predictor of wait time, our univariate analysis suggests that referral quality may significantly influence wait time. It is likely that our analysis was under powered and, therefore, referral quality may be a factor that influences wait time. This needs further investigation. Although referral quality was not a significant predictor of other patient outcomes, we found that wait time was a significant predictor of disease flares, IBD-related hospitalizations and additional referrals to IBD specialists for semi-urgent patients. This is a key finding, as it suggests that patients with longer wait times are more likely to experience poor outcomes, and that additional pressure is being placed on the health care system (i.e., additional referrals) because of prolonged wait time.

Previous literature has suggested that the quality of referrals to speciality care, including those for IBD specialists, are variable and often lack the information needed to diagnose and treat patients’ concerns (14). Research pertaining to the quality of referrals to IBD speciality programs supports the current study’s findings that the majority of referrals seeking speciality care for IBD patients are generally of low quality (14,20). We excluded several variables from our regression models (i.e., location, extent) given that the information on the referral was likely not reported accurately. Unlike other studies, which have evaluated GI referral quality to better understand patient demographics or disease history, this study’s univariate analysis suggests that poor quality referrals may adversely affect wait time; however, more research needs to be completed to understand this relationship. Understanding this relationship is an important step in understanding what solutions will most effectively address and improve excessive wait times for GI specialists (11,12).

### Impact for IBD Patients

Recognizing the widespread effect of referral quality on timely access to health care services and other patient outcomes is paramount to quality improvement within the health care system. Long wait times are problematic for both patients and their families, potentially resulting in decreased quality of life and disease exacerbations (9,11,20). Prolonged wait time may also cause additional stress for patients and families who feel like the process is out of their control, yet feel the need to advocate for themselves because of extensive wait times. As this study found, advocacy may take the form of additional referrals sent on behalf of the patient. We also found that prolonged wait time significantly increases disease flares and IBD-related hospitalizations which causes physical duress for the patient, as well as increased financial pressure on the patient and the health care system. This observation has previously been reported in the literature (21).

There are a number of other system-related costs associated with low-quality referrals in relation to health care system inefficiencies. For instance, if a referral is missing key information (i.e., diagnosis or information about extent/location), administration may request further information from the referring practitioner or request a new referral. This practice is costly in terms of the time inefficiency for administrative personnel and may prevent administration from scheduling new specialist appointments and draw on the limited time of the referring practitioner in order to address information gaps. If adequate referral information is not available for accurate triage, the patient may be subject to additional or redundant tests and procedures which can be costly for the health care system, as well as the individual who may experience additional financial costs associated with missed time from work, procedure and travel. Further, Jiwa et al. have suggested that specialists are less likely to schedule patients if they do not have confidence in the information provided by the referring practitioner (22). This is particularly problematic as the referring provider was the only significant predictor of wait time based on this study. Patients referred from general practitioners are more likely to have a prolonged wait time. Therefore, it is important that referring providers are providing high-quality referrals in order for their patients to be seen and to reduce system costs.

### Study Strengths and Limitations

The core strength of this study was the consistency of data collection from a single tertiary center and that all data were collected from one IBD speciality program, which ensured that there were no differences due to program-specific triage or referral processes. All referrals that were included in the analysis were semi-urgent referrals taken from a 1.5-year time period with little difference in program wait time over that time. The study design (i.e., development and use of a metric to measure referral quality) included similar variables as other GI studies, such as symptoms (i.e., abdominal pain), and evidence of diagnostic workup, which serves to validate our approach to the design of the quality metric (14,15,23).

Limitations include using only semi-urgent referrals in the multivariate regression analysis, which may limited the power to detect an association between referral quality and wait time in this study. The majority of people being referred were already diagnosed with IBD; therefore, the study may not represent patients who were referred but not yet diagnosed with IBD. Second, all referrals to the NSCIBD program were captured from one specialist’s list due to feasibility purposes given the lack of centralized IBD-specific triage system at the time the study was conducted. Patients were triaged by a group of gastroenterologists, depending on their rotation. This factor was examined as part of the analysis and was found to not have a significant effect on the results. Further, there are several electronic medical records that are being used within the provincial health authority, which means it is possible that information may have been missed (information bias). Further, we only noted whether or not a variable was included in the referral and did not explore the influence of the nature of the specific variable itself on the relationship between referral quality and wait time. Collecting this information would have strengthened our analysis and could help to better understand whether patients with certain disease-related characteristics experience longer wait times than others. Another potential limitation is that the metric designed to classify referral quality was designed based on the needs of the physicians and nurse practitioners of the NSCIBD program. The existing literature on GI referral quality overwhelmingly refers to referrals for dyspepsia or general GI issues, not specifically IBD; so, only a few other metrics were relevant for this project (14,15,23).

### Future Directions

Moving forward, it is crucial that we better understand how the referral process may impact access to services and overall patient quality of life. An intuitive first-step solution to address the quantity of low-quality referrals would be to develop and implement a mandatory standardized IBD referral form; however, we found that although a standard form was available, it was only used 7.5% of the time. Previous research also supports that standard forms are rarely used by referring providers (24). This study found that referring providers significantly influence patient wait time. If a standard form was to be developed, it would be important to include specific questions relating to IBD similar to those in the current quality metric and to clearly communicate expectations with referring providers.

Some programs have been experimenting with a number of strategies to combat long wait times without using a standard form. Peer review of referrals involves groups of general practitioners and specialists providing feedback to referring providers on their referrals. It has been shown to improve referring provider knowledge of what is needed based on to whom they are referring, as well as individual referring provider referral rates (25,26). However, there are indications that peer review can result in peer pressure and scrutiny concerning whether the referring provider is a good or poor clinician based on referral rates (27). Therefore, any peer review process should be undertaken with caution. Another strategy could involve implementation of standardized referral forms through a universal electronic referral system. Currently, in Nova Scotia, there are a number of referral systems, which has resulted in a fragmented communication and referral process. Amalgamating all referrals to a single system could facilitate the referral process and could improve referral quality by improving communication between the referring provider, specialist and, ideally, patient. Appropriate financial and human resource supports would need to be provided to system users in order for the system to be a success.

In Canada, it is virtually impossible for patients to access speciality health services without first receiving a referral. Although referrals can be useful for triaging patients who need urgent care and for providing patient history to specialists, this process can also be problematic due to missing information and subsequent costs to the health care system. Other strategies involve finding ways that reduce our reliance on referrals to medical doctors. For instance, walk-in clinics have been tested in the paediatric setting (28). However, walk-in clinics can be problematic due to lack of continuity of care and can place significant responsibility on patients for advocacy and vigilance. Further, long wait times can continue to grow if walk-in clinics frequently exceed their daily capacity. Another way of overcoming wait times may be through increased use of allied health professionals and nursing staff, including nurse practitioners. As physician shortages become an increasingly prevalent issue, it is imperative to shift our medical values to recognize the high quality of care that can be provided through integration of other health professionals in medical practice. By moving towards more collaborative and multidisciplinary practices, improvements to the way patients access and navigate the health care systems can be identified and implemented.

## CONCLUSION

Prolonged wait times are significantly associated with adverse IBD patient outcomes and increased costs for the patient and system. Although the majority of IBD patients rely on referrals in order to receive speciality care, it is evident that the current referral process is resulting in prolonged wait times. Moving forward, it is imperative that patients be seen sooner in order to improve patient outcomes and reduce systems costs. As a result, we need to critically reflect on the current referral system, and identify and implement the necessary changes to improve the patient experience.

## Notes

*Conflicts of Interest and Sources of Funding*: No conflicts of interest or sources of funding to report
